# Ten simple rules for finding and selecting R packages

**DOI:** 10.1371/journal.pcbi.1009884

**Published:** 2022-03-24

**Authors:** Caroline J. Wendt, G. Brooke Anderson

**Affiliations:** 1 Department of Statistics, Colorado State University, Fort Collins, Colorado, United States of America; 2 Department of Mathematics, Colorado State University, Fort Collins, Colorado, United States of America; 3 Department of Environmental & Radiological Health Sciences, Colorado State University, Fort Collins, Colorado, United States of America; Dassault Systemes BIOVIA, UNITED STATES

## Abstract

R is an increasingly preferred software environment for data analytics and statistical computing among scientists and practitioners. Packages markedly extend R’s utility and ameliorate inefficient solutions to data science problems. We outline 10 simple rules for finding relevant packages and determining which package is best for your desired use. We begin in Rule 1 with tips on how to consider your purpose, which will guide your search to follow, where, in Rule 2, you’ll learn best practices for finding and collecting options. Rules 3 and 4 will help you navigate packages’ profiles and explore the extent of their online resources, so that you can be confident in the quality of the package you choose and assured that you’ll be able to access support. In Rules 5 and 6, you’ll become familiar with how the R Community evaluates packages and learn how to assess the popularity and utility of packages for yourself. Rules 7 and 8 will teach you how to investigate and track package development processes, so you can further evaluate their merit. We end in Rules 9 and 10 with more hands-on approaches, which involve digging into package code.

## Introduction

The R programming language is an open source language that has become a dominant quantitative programming environment in academic data analysis, enabling researchers to share workflows and reexecute scripts within and across subsets of the scientific community [1]. As the R ecosystem—in which the life of modern data analysis thrives—rapidly evolves alongside the burgeoning R community, R is exhibiting sustained growth when compared to similar languages, especially in academia, healthcare, and government [2]. In particular, R is increasingly favored in computational biology and bioinformatics, two of many disciplines generating extensive, heterogeneous, and complex data wanting for heavy-duty data analysis tools that (ideally) support computational reproducibility [3,4].

R was developed by statisticians, and its base software is collaboratively maintained by an international group of core contributors [5]. The language has an ecosystem of extensions beyond this base software. Unlike several classic proprietary languages such as MATLAB and SAS, R users can extend the capabilities of the R language, then share their work with other users in a formalized way through extensions called packages (as a note, “package” is not perfectly synonymous with “extension” because some packages come with base R and technically aren’t extending the base software, but we’ll use “package” throughout as the commonly recognized colloquial term). An R package is a fundamental unit of shareable code.

These user-contributed packages comprise the bulk of enhancements made to the R environment [6]. In fact, much of what we know about statistical methods as well as data analysis and visualization is wrapped up in R packages—written and documented by R users from around the world. Are there R packages for wrangling and cleaning data frames, analyzing gene expression microarray data, or training regression and classification models? Yes! But how do you find an R package to help you assess the beta diversity of a population, perform dimensionality reduction, or design interactive applications and graphics? This answer is not as simple; there are tens of thousands of R packages. As a natural consequence of the open source nature of R, there is considerable variation in the quality of R packages and nontrivial differences among those that provide similar tools.

Packages are essential to venturing beyond base R and, thus, quickly become an integral aspect of advancing your R capabilities. Those who have used R packages may know that, although leveraging existing tools can be advantageous, it can be challenging to find a suitable package for a given task, which can obstruct potential benefits. An advanced R user—having developed an intuition for their workflow—may be relatively confident when searching for and selecting packages but may still occasionally discover gaps in their knowledge and approach. By contrast, newer R users who are unfamiliar with the structure and syntax of the language may struggle to discover packages because they do not know where to search, what to look for, or how to sift through options. When first learning R, and sometimes despite experience, 2 main obstacles are figuring out how to (1) find a package to accomplish a particular task or solve a problem of interest and (2) choose the best package to perform that task.

Yet, both newer and more experienced users don’t need to struggle alone to maneuver these obstacles—there is a science behind selection. We can inform our decisions by assessing, comparing, and filtering options based on indicators of quality such as utility, association, and reputation. In this paper, we outline 10 simple rules for finding and selecting R packages that offer something for everyone, so that you’ll spend less time searching for the right tools and more time coding.

## Rule 1: Consider your purpose

Usually, there are several ways to accomplish a task while programming, albeit some more elegant and efficient than others. Packages are great when a task has a broad or complex scope beyond what you can (or want to) attempt from scratch. While there are ways to develop an algorithm in base R, a relevant package may accomplish your goal with less code and fewer bugs. Before looking for a package to use for a task, however, determine whether you need one. Consider your purpose by first identifying your goal then defining the scope and tasks to achieve it. For some tasks and workflows, it is practical to code your own solution with base R or with packages you’re already using, while other tasks benefit from new tools.

If the scope of your task is simple or reasonable, given your knowledge and skills, using an R package may not be appropriate. There can be advantages of coding in base R to complete your task or solve your problem. First, when you code from scratch, you know precisely what you are running, and so your script may be easier to decipher and maintain over time. Conversely, packages require you to rely on shared code with features or underlying processes of which you may not be aware. Second, although base R is relatively stable and slow to change, many R packages evolve rapidly; changes made to packages can cause defects in portions of your code that can be hard to diagnose.

Extensive tasks justify sophisticated frameworks with several functions that form a cohesive package or even a suite of related packages. Data manipulation is one such common task that has been streamlined by packages such as data.table, dplyr, and tidyr [8–10], the latter two of which are part of the tidyverse suite (Table 1). Nevertheless, don’t be discouraged if you have a task that seems too unusual for a package. There are indeed packages for seemingly singular tasks, which you may favor over coding from scratch. You can use an R package to access the Twitter API, send emails from R, and there is even a package for converting English letters to numbers as on a telephone keypad [17–19].

**Table 1:** Common R packages for general purposes.

Package	Description	Year	Reference	Documentation
**Data Manipulation**				
readr	Read rectangular data (for instance, csv, tsv, and fwf)	2015	[7]	https://readr.tidyverse.org/
data.table	Perform data manipulation operations	2019	[8]	https://cran.r-project.org/web/packages/data.table/vignettes/datatable-intro.html
dplyr	Grammar of tidy data manipulation	2014	[9]	https://dplyr.tidyverse.org/
tidyr	Tidy messy data	2014	[10]	https://tidyr.tidyverse.org/
**Statistical Modeling**				
broom*	Tidy model output; convert statistical objects into tidy tibbles	2014	[11]	https://broom.tidymodels.org/
purrr	Functional programming tools for functions and vectors	2015	[12]	https://purrr.tidyverse.org/
caret	Framework for predictive modeling	2007	[13]	https://topepo.github.io/caret/index.html
**Data Visualization**				
ggplot2	System for data visualization	2007	[14]	https://ggplot2.tidyverse.org/
kableExtra	Build complex tables and manipulate styles	2017	[15]	https://haozhu233.github.io/kableExtra/
rmarkdown	Authoring framework for data science and reproducible research	2014	[16]	https://rmarkdown.rstudio.com/lesson-1.html

*See the biobroom analog in Bioconductor.

If you decide you need a package, next ask yourself: Which functionalities in base R are restrictive in context of your task? Which new functionalities would expand what you can do? If you identify limitations of your current toolbox before searching for new tools, you’ll be primed to recognize what you do and don’t need. List domain-specific keywords that describe what you are trying to do and how you could do it, so you narrow your search. Identify the type of inputs you have and envision working with them; contemplate the desired outputs and corresponding format. Suppose you are using Bioconductor packages (a set of packages focused on genomic and related data, further described in Rule 3) in analyses and have outputs you want to visualize; you must consider that the inputs may be of a certain class—a type of S4 object, for example (this is a type of object for storing data in R that is commonly used in, among others, Bioconductor packages and that can require different tools and approaches than the dataframe-centered approach that is now commonly the focus when teaching R to new users) [20]. When you look for a package to visualize the data, you’ll want to choose one that harmoniously handles this type of object as an input. If you don’t quite know (or have a difficult time articulating) what you need, don’t hesitate to search the internet for examples that capture your purpose. It can be helpful to search with Google (and Google Images for plots and figures) to identify the terminology that describes what you are trying to do, how people approach similar tasks, and potential challenges that may arise.

## Rule 2: Find and collect options

You’ll encounter tips and leads for finding R packages in various places online, in print, and elsewhere. If you’ve searched for packages a lot, you likely have a shortlist of tried and true starting points—a perk of being a long-time R user. On the other hand, new R users haven’t yet developed that internal compass, and they often don’t know where to start looking for a package to suit their task. Here are a few directions to head in: You can discover new packages any time you learn R-related topics, browse the internet, or connect with the international community of R users.

When learning how to program in R, you are typically introduced to some of the most common packages, which tend to have more general purposes. Table 1 gives some examples of popular packages you may have learned about in an introductory R course or book, particularly if it focused on the tidyverse approach to coding in R. In addition, reputable online tutorials, courses, and books are helpful resources for acquiring knowledge about packages that are versatile and reliable—they tend to be concise, accessible, and either affordable or offered at no cost to the learner. Codeacademy, Coursera, and other Massive Open Online Courses provide platforms for interactive learning. Numerous R programming books are available freely online, many of which are highlighted on the bookdown.org site or obtainable through RStudio’s books. R packages are also associated with printed content from scientific publishers. For example, Springer has a Use R! series with books covering specialized topics from finance to marketing, from chemometrics to brain imaging, and more. Additionally, consider reading titles from The R Series at Chapman & Hall/CRC. The books in this series span topics related to R in terms of its applications (for instance, epidemiology, engineering, social sciences), uses (for instance, statistical methods), and development (for instance, creating packages).

You can also browse online to look for R packages. Search engines such as Google return ample pages related to anything ". . . in R". This can be a good place to start, especially if you use the list of terms you developed for Rule 1. For a more directed approach, you can search the package lists on repositories including the Comprehensive R Archive Network (CRAN), Bioconductor, and rOpenSci. For example, you can search CRAN directly from the R command line using the pkg_search function from the pkgsearch package; in other words, to search for packages related to mixed models on CRAN, you could run: pkgsearch::pkg_search(query = "mixed models") [21] (note that here and throughout, we use this compact package::function() notation to indicate a function from a specific package). You can also browse packages available on code-sharing websites like GitHub and GitLab (see Rule 3), with language filters for finding repositories that heavily rely on R code.

To search with a more targeted approach, you can reference a curated list of R packages on a particular topic. CRAN Task Views concentrate on topics from certain disciplines or methodological approaches (for instance, Econometrics, Genetics, Optimization, Spatial). In the HTML version, you can browse alphabetized subcategories of packages within each Task View and read concise descriptions to find tools with specific functionality. Alternatively, you can access Task Views directly from the R console with ctv::CRAN.views() [22]. There are currently 41 Task Views that collectively contain thousands of packages, all curated and regularly tested. Moreover, CRAN Task Views provide tools that enable you to automatically install all packages within a targeted area of interest. Ultimately, by providing task-based organization, easy simultaneous installation of related packages, meta-information, ensured maintenance, and quality control, CRAN Task Views address several major user-end issues that have arisen due to the sheer quantity of available packages [22].

The computational biology and bioinformatics analogs of CRAN Task Views, BiocViews, are lists of packages available through the Bioconductor repository that provide tools for analyzing biological data. Your keywords from Rule 1 will come in handy here, too; for instance, you can search “RNA-seq” to find all packages related to high-throughput sequencing methods for analyzing RNA populations. You can also browse the BiocViews tree menu to narrow your search based on which general and specific categories describe your task. The list includes each package’s name linked to information and documentation, its maintainer, a short description, and its rank relative to other Bioconductor packages.

Other curated collections and topically-linked lists of packages are also available. CRANberries, for example, is a hub of information about new, updated, and removed packages from CRAN. Another place to find well-maintained tools is rOpenSci packages. These filterable and searchable peer-reviewed (see Rule 6) R packages are organized by name, maintainer, description, and status markers based on activity, association, and review.

You’ll also find tips on R packages by joining in R’s vibrant international community. An inclusive and collaborative community is an overlooked, yet integral, aspect of a software’s success [23]. The R community has a widespread internet presence across various platforms. Members are markedly active on Twitter, a place where R users seek help, share ideas, and stay informed on #rstats happenings, including releases of new packages [24]. R-related blogs such as R Weekly and R Views serve as another accessible, informal, up-to-date, and more detailed avenue for tracking, communicating, and promoting R-related information [25]. For example, Joseph Rickert, Ambassador at Large for RStudio, writes monthly posts on the R Views blog highlighting interesting new R packages [26]. Rickert also features special articles about recently released packages and lists of top packages within certain categories, including Computational Methods, Data, Machine Learning, Medicine, Science, Statistics, Time Series, Utilities, and Visualization. Other blogs are available that give updates on specific packages or collections of packages, like the tidyverse blog, which covers packages in the popular tidyverse suite.

Members of the R community gather regularly at conferences to share their latest work. You can attend—or access content from—these conferences to learn about improved, recently released, or up-and-coming R package developments and applications. Two large annual R conferences with broad scopes are rstudio::conf and useR!. There are also a number of smaller, more discipline-tailored R conferences, including R/Pharma (pharmaceutical development), R/Finance (applied finance), and BioC (open source software for bioinformatics). Conferences in your field may foster connections with fellow scientists who use R for similar tasks and help you collect information about packages related to your expertise. Talks and presentations at conferences are often recorded and made available online for playback if you can’t attend in person or wish to revisit content from past conferences. For instance, RStudio has a webpage devoted to videos of past conferences by year and the talks from useR! are posted on the R Consortium YouTube channel. The R community and rOpenSci have also popularized nontraditional rOpenSci Unconferences and R Collaboratives at which people interact and share ideas in a collegial and unstructured atmosphere; as you may imagine, R packages are a hot topic of conversation at these events.

Don’t forget, a developer of an R package may intend for it to be private (exclusively for personal or professional use) or public (at no cost and available for use by anyone) [27]. If your task is specific to a line of research, consult colleagues to see if they have a relevant (private) package they’d be willing to share.

## Rule 3: Check how it’s shared

R developers share their packages through several platforms. The primary type of platform is a repository, where developers share packages for public use. An R package repository provides a central online location in which packages are stored and managed. Some repositories provide vetting mechanisms that tame unwieldy aspects of the R ecosystem by regularly checking underlying code and managing corresponding webs of dependencies. Two traditional repositories for R packages are CRAN and Bioconductor; however, there are lesser-known repositories to explore. Developers can also share their R packages through large code-sharing sites, including public version control platforms like GitHub and GitLab, which have fewer restrictions on the format or content of shared code compared to repositories. For more private sharing, rather than making packages accessible to everyone via internet repositories, some developers share their code in zipped files directly with collaborators (see Rule 2).

The CRAN repository is the most established R package repository, and it serves as the primary source from which you’ll likely install R packages. The Institute for Statistics and Mathematics of Wirtschaftsuniversität Wien hosts the CRAN server and Contributors from the R Core Team (established in 1997) maintain its monumental collection of R packages (about 18,000 as of August 2021) on almost any conceivable topic, from random forests to multidimensional maps [5,28,29]. Due to its longevity and historical role, Rickert asserts that “CRAN is the greatest open source repository for statistical computing knowledge in the world” [27]. A key advantage of using a package from CRAN is that the repository integrates easily with your base R installation [30]. You can simply use the install.packages() function with default values to install a package from CRAN, along with all of the packages it depends on; source and/or binary code are automatically saved to your computer in a designated package library [5,31]. When you want to use a particular package, you load it to your R session via the library() function from base R [5]. These features ensure that integration of a new package with your version of the base R software is typically silent and seamless.

CRAN imposes some regulations on the submission and maintenance of its hosted packages. For example, a package must pass a series of initial stability tests in accordance with the CRAN Repository Policy before obtaining publication privileges [32]. CRAN does not, however, have a peer review process (see Rule 6), so these checks do not extend to the substance of the package, in terms of ensuring it works as advertised in the documentation. At worst, then, a CRAN package might have a solid framework with help files for each outward-facing function, but its underlying code might not necessarily do what it claims. CRAN maintainers actively monitor contributed packages to ensure compatibility with the latest version of R and dependencies; they update metadata accordingly and ask for modifications or remove packages that don’t uphold these publication quality standards. For instance, to see the metadata page for the ggplot2 package, you can go to https://CRAN.R-project.org/package=ggplot2 [14]. Replace the package name at the end of the web address with the name of any other CRAN package to access analogous package-specific metadata.

If you’re looking for tools specifically designed for high-throughput genomic data, the Bioconductor repository is a more specialized repository than CRAN that’s worth investigating [33]. The Bioconductor project was motivated by a need for transparent, reproducible, and efficient software in computational biology and bioinformatics; it supports the integration of computational rigor and reproducibility in research on biological processes [3]. Bioconductor packages facilitate the analysis and comprehension of biological data and help users solve problems that arise when working with high-throughput genomic data such as those related to microarrays, sequencing, flow cytometry, mass spectrometry, and image analysis [33]. Bioconductor boasts a modularized design, wherein data structures, functions, and the packages that contain them have distinct roles that are accompanied by comprehensive documentation. For example, the page for the package DESeq2 can be found at https://bioconductor.org/packages/release/bioc/html/DESeq2.html; you can view pages corresponding to other Bioconductor packages by altering the URL as described [34]. Accessibility is a pillar of the Bioconductor project: All forms of documentation, including courses, vignettes, and interactive documents, are curated for individuals with expertise in adjacent disciplines and minimal experience with R (see Rule 4) [3]. Bioconductor packages also integrate well with base R; you should use BiocManager::install() to install Bioconductor packages with their corresponding dependencies [35].

Bioconductor has a structured release schedule and thus a unique package management system that automatically loads your Bioconductor packages in versions that are all from the same release cycle and compatible with your current version of R [33]. This helps, among other things, keep metadata properly aligned with sample data. Bioconductor has strict criteria for package submissions: The developer is bound to providing active user support over the years, while the package itself must be relevant to high-throughput genomic analysis, interoperable with other Bioconductor packages, well-documented, and comply with additional package guidelines [36]. To ensure Bioconductor software maintains a centralized content focus, their packages are coordinated and peer-reviewed. The peer review processes includes a technically focused review by a primary reviewer and may also include secondary reviews by content matter experts [33].

In addition to CRAN and Bioconductor, there are other smaller, more specialized repositories that share R packages. The nonprofit organization, rOpenSci, for example, runs a repository as part of their commitment to promote open science practices through technical and social infrastructure for the R community [37]. The repository only includes packages that have either been developed by staff to fill a gap in infrastructure or have passed their open review process. These packages are within the scope and aims defined by rOpenSci, including those that focus on data extraction, workflow automation, and field or laboratory reproducibility tools [38]. At a smaller scale, R package maintainers can create and host their own personalized repository using the drat (Drat R Archive Template) package, which enables developers to design individual repositories and suites of coordinated repositories for packages that are stored in and/or distributed through platforms like GitHub [39]. In some cases, this might be done to host a package that doesn’t meet certain restrictions from other repositories (for instance, to share data through a package that’s larger than CRAN’s size limit for packages [40]. There are other platforms strictly for the development, rather than distribution, of R packages such as R-Forge and Omegahat, which are beyond the scope of this paper [41,42].

Packages can also be hosted on online code sharing sites like GitHub or GitLab, both of which are online hosting platforms for repositories tracked via git version control. While there are evident benefits of exclusive R package repositories, there are also considerable advantages to hosting a package on these online platforms [27,43]. First, package developers can post the directory with all source code for their package as they develop it, from the earliest stages, rather than waiting until the package is publication-ready. What’s more, these platforms allow for continuous integration and continuous deployment for testing data and code to ensure a stable end-product.

One particularly popular and effective platform for sharing R packages is GitHub. An increasing number of packages are hosted on GitHub during the development stages—it will often be the first place you can find up-and-coming packages (see Rule 2). If stable release versions of these packages are published on CRAN or Bioconductor [44], GitHub is still a good place to explore planned changes for the next version that the developer will soon release. GitHub provides R users with open access to package code, a timeline of help resources (see Rule 4), and a direct line of communication to developers. Both R users and package developers benefit from interactive feedback channels through GitHub Issues and the Star rating system.

It’s straightforward to install the latest version of a package you want to use from GitHub via devtools::install_github("tidyverse/ggplot2") (here and in other code examples involving package names, we’ll use the ggplot2 package as an example). However, the decentralized nature of GitHub isn’t conducive to a tool that automatically locates and installs corresponding dependencies that are not on CRAN [45]. In fact, the rapid uptick in package development and ensuing inter-repository dependencies has sparked an ongoing debate on whether regulated repositories such as CRAN and Bioconductor are preferable to distribution platforms like GitHub due to this complication [27,43,46]. Further, when a package is installed from GitHub, typically only the source code is available, not binaries prebuilt for your computers’ operating systems. This means that the installation process will need to build the package from source on your computer, which may require certain compilers and other tools (for instance, XCode for macOS, Rtools for Windows).

When you’re searching for and selecting packages, it’s important to note that a single package is often available in multiple places. It’s best to start by checking formal repositories before code sharing sites. Packages that are on CRAN, Bioconductor, and/or rOpenSci are also usually on GitHub. There’s an unofficial, read-only GitHub mirror at CRAN, METACRAN, on which all CRAN packages have their own GitHub repositories with all versions, original dates, and authors. Additionally, package developers frequently use their personal GitHub accounts to publicize a package while it’s being developed and continue to host development versions after it’s been published on formal repositories such as CRAN. In this case, CRAN would host a “stable” version of the package, which may simultaneously be in the “master” branch of the developer’s GitHub repository for the package; forthcoming package updates would remain in other branches (for instance, a “development” branch) in the same GitHub repository. Although less relevant to new R users, this means that you can access and test upcoming versions of a package through GitHub, which may be particularly helpful if you’re developing a package with dependencies and want to know if upcoming changes to a package that your code depends on will cause issues. Lastly, there are some instances where a packages is posted on CRAN, then also published in a specialized repository. As part of their review process, for example, rOpenSci asks authors of submitted packages if they plan to publish on CRAN or Bioconductor, then packages often become available in both places.

## Rule 4: Explore the availability and quality of help

Current sources of information related to R packages are dispersed and plentiful. On one hand, this allows users to explore diverse solutions and discover new tools; on the other, not knowing where to find help can lead to inefficient and ineffectual roundabouts. Not all package resources share the same level of quality and the fact that there are many resources in aggregate does not imply that every package is associated with the same availability of resources.

Though all published R packages meet some minimal standard of documentation, beginners and users of complex packages will often want help documentation that is more thorough than this minimum. Help associated with R packages generally falls into one of 2 categories: help documentation that comes embedded in the source code of the package (i.e., ships with the package) and documentation or other resources that are available more broadly, beyond the package’s source code. The documentation that ships with the package can consist of help files, vignettes, README files, tutorials, and pkgdown documentation [47], and even code comments embedded in the R scripts that define package functions. Broader help resources can include webpages or online books whose content is hosted outside the package source, online tutorials, electronic mailing lists, and large and vocal communities of R users. Implicitly, if an author has expended time and effort to provide copious and useful help documentation—within package code, outside of it, or both—beyond the utility of the resources themselves, this suggests that the authors are serious about their package development and adds confidence that they know what they (and their package) are doing. Exemplary documentation can signify an exceptional package, while you might keep looking if the quality and quantity of help resources for a package is lacking.

At the most granular level, every R package should come with a help file for each outward-facing function—that is, functions which the package maintainer intends for you to use directly (as opposed to smaller help functions meant solely for use within other package functions). This practice is required for packages on some repositories, like CRAN, but might not be adopted for those posted on less restrictive platforms, like GitHub. For each outward-facing function in a package, once you’ve loaded the package in your R session, you can access its help file from the console via the help function or by typing? followed by the function’s name (for instance, help (mean) or? mean returns the help file for the mean function). Function help files provide information about how to use the function, including the parameters that can be set, the format to use when specifying arguments for these parameters, a description of the file, and even references to papers or other resources on which the function’s underlying algorithm was based. They often contain executable examples to help you get a feel for how to use the function in practice. If you’d like to check all help documentation for a package, you can access an index of help pages from the console via a call like help (package = "ggplot2") [5]. The documentation that accompanies functions within packages is critical; the fact that anyone can read it anytime and use it to guide their own work facilitates extensibility. The extent and quality of content in these help files will vary; ideally, packages should have thorough documentation at the function level. If the help pages alone leave you wanting, it may be less likely that the package has further (quality) documentation and therefore you might reconsider whether that package should be your first choice, if comparable options exist. In short, if the developer cannot initially communicate how their tool works, then you probably won’t want to use it.

Fortunately, plenty of R packages include additional documentation beyond this minimum. While the function-level help files can be rather technical and extraneous to new users, a vignette is a practical type of documentation in the form of a tutorial. A vignette is a detailed, long-form document that describes the problems an R package can solve, then illustrates applications through clear examples of code with coordination of functions and explanations of outcomes. See, for example, the extensive vignette for the sf package [48]. Packages can have multiple vignettes; you can view or edit a specific vignette or obtain a list of all vignettes for a package via the vignette() function from base R [5]. For a package shared through CRAN or Bioconductor, a list of the package’s vignettes can also be found on the respective homepage for the package. Particularly for packages hosted on GitHub, developers often create a mini-vignette in the README to provide a high-level overview of their package and its components, along with a basic introduction and short tutorial for its usage. This mini-vignette, displayed on the package repository’s main page, should simply tell you why you should use it, how to use it, and how to install it [31].

Interactive tutorials, most commonly those powered by the learnr package, are another effective and popular way to facilitate learning about R packages [49]. They’re either conceptual and exploratory or specific and structured in their content and can include a range of features: narratives, figures, videos, illustrations, equations, code exercises, quizzes, and interactive Shiny elements. You can run the learnr::run_tutorial() function to deploy the learnr tutorial included within a package, if one exists [49]. Alternatively, as of RStudio v1.3, you can access learnr tutorials for packages you’ve installed directly from the Tutorial pane in the IDE [50]. Some developers publish their learnr tutorials as Shiny apps, which can be called from within the learnr::run_tutorial function and accessed outside of the RStudio interface [49].

Several thousand developers have been using pkgdown to create static HTML documentation [47,51]. This tool allows developers to collect dispersed forms of documentation for their package such as help files and vignettes, then build a single package website with this information plus more. The pkgdown website itself as well as those for naniar, valr, and TransPhylo are examples of pkgdown documentation in action for R packages [47,52–54]. Sometimes, you can find a link to a package’s corresponding pkgdown website in the “URL” section of its CRAN page, which is often listed alongside its GitHub link.

So far, we’ve only mentioned types of help documentation that are incorporated in the source code of a package; they “ship” with the package and so you can investigate them by looking through the underlying package code. However, there are easier avenues for accessing help documentation encoded in a package. RDocumentation, for example, is a searchable website, package (install.packages("RDocumentation")), and JSON API for obtaining integrated documentation for packages that are on CRAN, Bioconductor, and GitHub [5,55]. RDocumentation may include an overview, installation instructions, examples of usage, functions, guides, and vignettes. This is a subsidiary reason why it may be useful to look for packages shared on these platforms (see Rule 3).

Some packages have a comprehensive set of resources beyond those encoded in the package source code. For instance, as mentioned in Rule 2, there are online and/or print books associated with certain packages. One popular method that developers use to publish books about their package is through bookdown, an extension of R Markdown that’s structured to integrate code, text, links, graphics, videos, and other content in a format that can be published as a free, open, interactive, and downloadable online book [56]. The bookdown package itself has an online book that details usage of the package [56,57].

Many other forms of help documentation can be shared outside of the package’s code. Some packages have corresponding online courses, which might be taught by the maintainer. GAMs in R, for instance, is a free interactive course on Generalized Additive Models in R with the mgcv package [58]. Every now and then, an online course will cover one or a few select packages that revolve around a central theme such as a types of modeling. A suitable example, Supervised Machine Learning Case Studies in R, features several tidy tools with a primary focus on tidymodels [59]. Galleries, as the name would imply, are sites that display works, articles, and/or code examples associated with certain packages. As you might expect, galleries can be valuable sources of inspiration, particularly for packages that provide tools for data visualization tasks. See, for example, The R Graph Gallery as well as the galleries for ggplot2 extensions, dygraphs for R, shiny, and htmlwidgets for organized displays of outputs from these packages [14,60,61]. The Rcpp gallery is an instance of a gallery that contains collections of articles and code examples for the Rcpp package [62]. Cheatsheets provide concise usage information for popular packages through code and graphics organized by purpose. RStudio hosts a number of these, including cheatsheets made by RStudio’s team and those contributed by others. These cheatsheets can be accessed directly via the RStudio Menu (Help > Cheatsheets) or from the RStudio website on which you can subscribe to cheatsheet updates and find translated versions. Packages with their own cheatsheets include caret (statisical modeling and machine learning), data.table (data manipulation), devtools (package development), dplyr (data transformation), ggplot2 (data visualization), leaflet (interactive maps), randomizr (random assignment and sampling), sf (tools for spatial data), stringr (character string manipulation), and survminer (survival plots) [8,9,13,14,45,48,63–66]. Further information about some R packages is available in video tutorials, webinars, and code demonstrations.

There are also resources that involve asking for help, and you can size up the presence of the package you’re considering through these resources, as well. The R-help mailing list, to which you can subscribe and send questions, is moderated by the R Core Development Team and includes additional facets for major announcements about the development of R and availability of new code (R-announce) and new or enhanced contributed packages (R-packages) [67]. In the early days, R-help was the only way to seek direct assistance outside of knowing and asking other R users personally; since, the R community (for instance, RStudio Community, Bioconductor Support) has evolved to make asking for help more widespread and accessible, with inclusion and creative problem-solving as hallmarks of its online presence [68]. Certain packages have independent mailing lists; statnet is an example of a suite of packages that has its own mailing list [69].

If a package has a development repository on GitHub, check the Issues to verify that the maintainer is responsive to posts and fixes bugs in a timely manner. In addition, you can search discussion forums such as Stack Overflow, Cross Validated, and Talk Stats to assess the activity associated with the package in question. Analyses of the popularity of comparable data analysis software in email and discussion traffic suggest that R is rapidly becoming more prevalent and is the leading language by these metrics [2,70]. When you encounter a problem, it’s good practice to first update the package to see if the trouble is due to a bug in a previous version—if the problem persists, seek help by finding or posting a reproducible example [71].

As part of learning to assess the help resources for any package, it’s helpful to reference a very well-documented package. One example of a package—mentioned a few times already—with notable documentation and first-rate help resources is Rcpp [62]. The developers maintain both a main and additional website with a wealth of organized information about the package and resources, including a gallery of examples, associations, publications, articles, blogs, code, books, talks, and links to other resources with Rcpp tags. The package has a dedicated mailing list, for developers using the package. Rcpp is also thoroughly described in a print book by its maintainer [72] and in chapters or sections of other books (for instance, [71]).

## Rule 5: Quantify how established it is

Consulting data to inform comparisons is never a bad idea; numerical data associated with R packages will give you an impression of how popular a tool is and whether it’s stood the test of time. There are metrics that you can use to determine how often (and recently) an R package has been updated through new versions, how long it’s been around, and how often it’s been downloaded.

You can find several of these metrics by visiting the site(s) on which a package is shared (see Rule 3). If a package is posted on CRAN or Bioconductor, its page on that site will include the date on which its latest version was released as well as a link to “Old sources”—previous versions of the package and the release date of each. The page also provides a link to a NEWS file, where you can see a list of all published versions as well as the major changes made from one version to the next. All R packages shared through CRAN or Bioconductor must be versioned, and many adhere to a versioning scheme of the form major.minor.patch, where each placeholder is a nonnegative integer (for instance, 1.7.9). The major version number increases with larger or incompatible API changes, the minor version number increases with backwards compatible functionality changes to be released, and the patch version number increases with smaller backwards compatible bug fixes and new features. Overall, version numbers are typically assigned and incremented based on standardized rules to signal code changes and modifications to software packages over time and to distinguish developer versions from release versions. So to further determine if a package is well established in the R community, refer to its number of versions and updates (more is better) in addition to the date of the most recent versions and updates (newer is typically better, as an indication of active maintenance, although in some cases a package with a stable algorithm and few dependencies may not need many updates).

You can also see, on a package’s CRAN or GitHub page, the number of reverse dependencies it has. A reverse dependency is to an R package as a citation is to a scientific paper. If you are a developer of a package, reverse dependencies are all the packages that depend on your package, just like if you are a researcher, citations are all the published papers that have cited your published paper. Because there are fewer R packages for the same task compared to papers supporting the same claim, a reverse dependency is perhaps more important to a package than a citation is to a paper. When a package has numerous reverse dependencies, the code underlying many other R packages incorporates functions from that package, which can indicate that other R developers find that package useful.

If a package is shared through a GitHub repository, the homepage for the repository will list the number of GitHub Stars, Forks, and Watchers for that particular repository. As proxies for a repository’s overall following—and possibly indirect measures of quality and impact—Stars indicate that people like or are interested in a project, Forks reveal the number times a repository has been copied, and Watchers represent GitHub users who are subscribed to updates on a repository’s activity [73]. Although not a definitively unbiased metric, a large number of Stars, Forks, and Watchers associated with a package implies a substantial following and widespread usage [74].

RDocumentation (see Rule 4) is rich with stats on R packages, uniting many of the previously discussed metrics, while also providing cross-package summaries. RDocumentation hosts a live Leaderboard with trends including the number of indexed packages and indexed functions, most downloaded packages, most active maintainers, newest packages, and newest updates. What’s more, each package is assigned a percentile rank—featured on its RDocumentation page—that quantifies the number of times a package has been downloaded in a given month. A ranking algorithm computes the direct, user-requested monthly downloads by accounting for reverse dependencies (indirect downloads) so packages that are commonly used within other packages, and hence frequently downloaded as automatic downloads with those packages, don’t skew the calculation [75]. You can research stats on corresponding dependencies for a more holistic picture. The dlstats package also has useful tools for exploring metrics for R packages [76].

Most of the aforementioned resources will help you learn more about packages on an individual basis. Wouldn’t it be nice if you could compare related packages that may have features that overlap? Some clever R users at an rOpenSci Unconference thought so, too, and developed a package called packagemetrics: A Package for Helping You Choose Which Package to Use [77,78]. You can install packagemetrics via devtools::install_github("ropenscilabs/packagemetrics") and run, for example, packagemetrics::package_list_metrics(c("ggplot2")) [45,77]. Then, you can use packagemetrics::metrics_table() to obtain a table of information that includes each package’s respective number of downloads, GitHub Star rating, tidyverse compatibility, and several more indicators to help you decide which package is most established and widely used [77,79].

An important caveat to consider: Several of the metrics introduced in this Rule are based on how often a package is downloaded; however, it’s not uncommon for users to download a package to explore its functionality and ultimately decide against using it for their task. Therefore, these suggested metrics can provide a first pass at identifying popular packages—and will, in particular, help you distinguish popular packages for common general tasks, like data wrangling and visualization—but may be less helpful when you’re selecting a package for a very specialized task. In this case, evidence of peer acceptance and review will usually be more instructive, which brings us to our next Rule.

## Rule 6: Seek evidence of peer acceptance and review

Peer review is an important aspect of scientific research, not least because it establishes scholarly credibility. While scientific articles undergo a peer review process that’s fairly standardized across disciplines, R packages have a broader range of possibilities for peer review or other evidence of acceptance by members of the scientific community. You can research information about an R package in different forms of literature and determine the extent to which it’s been validated by the scientific community.

Some maintainers of select packages have written an article describing their package and its algorithms. These articles must go through the traditional peer review process in order to be published. Certain journals publish articles about R packages themselves, whereas others feature work that used a particular package. Journals with a targeted and technical focus on R packages and other open source scientific software include the Journal of Open Source Software (JOSS), the Journal of Statistical Software, and The R Journal. Discipline-specific journals like BMC Bioinformatics may also publish articles to introduce R packages that have proven to be useful for aspects of the bioinformatics research process, for example. Try searching various websites for prominent journals in your field with queries that include "R package" to find these types of articles. The number of Google Scholar citations for a package or, similarly, an article about a package, can also help serve as a metric for a package’s impact on scientific research and utility in research contexts (see Rule 5).

For an R package featured in a peer-reviewed journal article, you can be assured that the ideas and principles behind the package have been evaluated by outside reviewers. However, such a review may not have included a fine-grained peer review of the underlying code in the package itself. There’s an expanding movement toward direct peer review of R packages and other open source scientific software at the code level. More specifically, rOpenSci is a unique example of an ecosystem of open source tools with peer reviewed R packages (see Rule 3) [37]. The rOpenSci organization oversees peer review of R packages at a highly detailed level that’s increasingly desired by scientists; packages published through their repository have passed a rigorous external review of the code. JOSS has a review process modeled after rOpenSci’s approach [80]. While JOSS exclusively publishes brief papers about software packages (code and documentation) that support research in a scientific context, rOpenSci limits their scope to packages that help scientists implement reproducible research practices and manage the data life cycle [38,80]. Each of their review processes are conducted openly on GitHub, and, as a result, the scientific community can view the discussions between package maintainers and peer reviewers that form this transparent process.

You can also seek evidence of a package’s peer acceptance by determining how often researchers have used it within scientific papers describing research, rather than package-focused papers. Occasionally, useful R packages may be casually mentioned in the Methods section of a publication, but it’s best practice for researchers to formally cite R packages in the References section. Packages hosted on CRAN or Bioconductor have standardized citation formats, and a call like citation("ggplot2") will print the suggested citation for the package [5]. In response to the rising number of researchers creating tools and software to work with their data, GitHub has granted developers the ability to obtain a digital object identifier (DOI) for any GitHub repository archive so that software and code can be cited in academic literature [81]. These citation formats and DOIs enable networks of research associated with packages to form, and you can explore these networks. If you search a package directly by name in Google Scholar, the Cited by link displays the number of times it’s been cited. Clicking the link will allow you to see all the publications that have formally cited the package.

## Rule 7: Find out who developed it

There’s variation in R users’ backgrounds and skills, and the same is true for R developers. That said, the R community prides itself on embracing newcomers at all levels of involvement. This Rule doesn’t imply that worthwhile packages are exclusively written by well-known authors. Rather, associations and reputation can be a proxy for quality; in this way, the process of evaluating and comparing R packages is no different than other decisions. In fact, as you become more immersed in the R community, you’ll find that name recognition is a factor, among many, that helps you establish trust in certain tools more quickly than others [74].

Information about the package authors can be found in the metadata of the package. If you’re looking at the source code of the package, there should always be a DESCRIPTION file with metadata. The DESCRIPTION is a succinct record of the package’s purpose, dependencies, version, date, license, associations, authors, and other technical details. In the “Author” field of this file, you can identify both the “Maintainer” of the package (a similar role to that of the corresponding author for an article) as well as other authors or contributors. For packages hosted solely on a platform like GitHub, this might be the only way to discover a package’s main author, but profile links for all authors are available under the Contributors section on the package’s main GitHub page. On the other hand, for packages hosted on repositories, this package data is usually also posted by the repository somewhere online. Packages on CRAN have a page of metadata that’s accessible through a standard web address (see Rule 3). Here, you might notice ORCiD links and/or various abbreviations associated with the names in the “Author” and Maintainer" fields that denote roles such as author [aut], creator/maintainer [cre], contributor [ctb], and funder [fnd]. Similarly, Bioconductor packages each have their own webpage, with some metadata at the top of the page, including the maintainer and other authors along with their roles (see Rule 3).

You can assess the credibility and commitment of R package developers through direct and indirect signals. Who made the package? Consider whether expertise in a certain domain is vital to the design and creation of the tool. Research the authors’ associations in academia, government, and/or industry and gauge the extent to which they have a primary role in R development. Sometimes, it’s possible to deduce author affiliations by simply looking at their domain name on their email addresses. Affiliations may serve as a proxy for package quality and sophistication insofar as standards, on average, tend to be higher in regulated settings. R packages produced by the United States Geological Survey (USGS), for instance, are maintained by domain experts that know field-specific data sources and algorithms very well. Moreover, authors with affiliations that include R package development as a paid responsibility (for instance, USGS, RStudio) might, on average, produce better packages. For a package hosted on GitHub, it’s worth noting whether it’s associated with a formal group, lab, or company account as opposed to an individual’s account, as the former might be more regularly maintained. If a package was developed by multiple authors, research each of them to evaluate the robustness of the team. You can learn more about their experience, active contributions to the R community, and history related to package development by exploring their profiles on GitHub, Google Scholar, Research Gate, Twitter, or personal or package websites. Frequent commits and effective resolutions of GitHub Issues can be a testament to the authors’ priorities and commitment. If an author has a history of package development, peruse their portfolio of packages to see if any are highly regarded or recognizable at first glance. You can even use functions from the cranly package to visualize the network of developers with whom the package authors cooperate [82]. If, in the past, you found a package that you like to use and is helpful for your work, it can be worthwhile to research other packages made by that author and/or maintainer. You might benefit from making a habit of paying attention to who makes tools that work well for your needs and are designed in a way that seems intuitive to you—take note of these “favorite” package authors.

As you may know, the R community values collaboration—it’s not uncommon for packages to be created and maintained by more than one person and, in many cases, large teams. Roles within these teams can shift as the package evolves. As an example, ggplot2 has a large team of authors and its original maintainer isn’t the same as its current one [14]. Oftentimes, existence of a large team can give you clues (although not guaranteed) about how the package is evolving in the R ecosystem. For one, it could indicate that a package is very useful and has generated rich community involvement, as is the case for ggplot2—many of its authors were added after the first release [14]. This might reveal that a package is part of a larger-scale and more organized project. Also, a sizeable team could imply that the package grew out of a substantial collaboration, like a funded grant with a multiperson team, which happens a lot for specific methods packages, like the flowCore package [83,84]. This might suggest that a package has a certain level of review and investment from several R programmers (the authors). It’s also useful to examine the full list of authors (even if it’s long) because you might not recognize the maintainer’s name, but you might recognize another author’s name. This can happen if the maintainer is a student or someone else that’s up-and-coming; sometimes, more senior authors don’t assume the role of maintainer, even though they’re quite invested in the project. It’s worth looking to see, for example, if someone who is well known for excellence in methods development is one of the nonmaintaining authors, which may help you gauge the quality of the fundamental algorithms and techniques in the package code.

## Rule 8: See how it’s developed

You don’t need to be a software engineer to identify strong package development. Scientific software developers sometimes neglect best practices; indeed, these shortcomings are evident in the tools they create [85]. There are concrete ways to measure a tool’s robustness beyond whether it works for those who didn’t create it. As a user, there are 4 main relevant package development protocols, in addition to some related indicators, that you can investigate to assess the underlying stability and utility of a package: dependencies, unit testing, version control, and continuous integration.

R packages often depend on other R packages; you should check the reputations of such dependencies when selecting a package, as quality packages will rely on a solid web of quality packages. What’s more, like other types of software, well-maintained R packages have multiple versions corresponding to iterative releases to indicate that the package is compatible with dependencies and loyally updated (for instance, bug fixes, general improvements, new functionality) [31,86]. You can explore the version history of a package to see if it’s up to date (see Rule 5). Package dependencies are not only important to establish stability, but might also give you a hint as to whether a package will fit well into your workflow. This is an especially critical consideration if you’re using a “tidy” workflow or working with special object types in, for example, a Bioconductor workflow [79]. You can review the list of dependencies for a package in question to check if it depends on some or many of the packages in your workflow. If this is the case, you can be fairly confident that the package uses the same (or at least similar) style and conventions as those you want to use.

A responsible developer with a consistent and reproducible workflow will implement formal testing on their code to examine expected behavior via an automated process called unit testing [31,87]. This helps assure the developer (and you) that the package’s underlying units of code do what they intend to do. The developer—and by extension, the package user—will benefit from unit testing, which results in fewer bugs, a well-designed code structure, an efficient workflow, and robust code that’s not sensitive to major changes in the future [31]. To alleviate the burdens of unit testing, testthat is a popular, integrative R package that helps developers create reliable functions, minimize error, and visualize progress through automatic code testing [88]. Developers are also interested in quantifying the amount of code in their package that has been tested. Test coverage, a measurement of the proportion of code that has undergone unit testing, is an objective metric for package developers, contributors, and users to evaluate code quality, and information about coverage can often be found as a badge (described further below) on its GitHub page. A high level of coverage is ideal—for example, ROpenSci recommends at least 75% code coverage for packages submitted to it for peer review [38]. However, there is not a single number that would make sense as a standard for every project, given differences in package aims and design. Many developers use the covr package to generate reports and determine the magnitude of coverage on the function, script, and package levels [89]. You can look at the source code for a package to confirm it has a testing suite; for instance, if you’re on a package’s GitHub page, you can navigate to the main Code page and look for a folder labeled tests or something similar.

Relatedly, developers who host their packages on GitHub often post status badges in the overview (README) section of the repository webpage for the master branch. GitHub badges are a common self-imposed method to signal use of best practices and motivate developers to produce software that’s high in quality and transparency [90]. They’re easily visible, interactive, and increase readability of the README. You might see, for example, license (license), version (release), dependency (dependencies), code coverage (codecov), or build status (build) badges, all of which are good indicators of package caliber. What’s more, badges have dynamic status tags to indicate the current status of the particular part of a workflow to which the badge refers. These tags are often colored on a spectrum to signal the relative meaning behind their values (for instance, red for bad, yellow for good, green for great), which sometimes depend on the baselines set by the developer. As an example, a build badge with a green passing status tag could be telling you that the package is passing continuous integration (CI) tests (CI is explained later in this Rule) [73]. Badges are also clickable buttons that link to a page that describes and displays detailed aspects of the metric associated with the badge. For instance, when you click on a codecov badge, you’ll likely end up on a page with a timeline, graphics, and information about the percentage and distribution of test coverage for the package. You can refer to special badges like CRAN, Bioconductor, or rOpenSci to determine which repository (or repositories) a package is shared on (see Rule 3).

As we mentioned in Rule 3, version control has an essential role in package development and computational literacy more broadly [31,91]. Version control is like a time capsule for your workflow because it monitors and tracks changes to files as a project evolves and stores them as previous versions to be recovered if necessary. In the words of Dr. Greg Wilson, “version control is as fundamental to programming as accurate notes about lab procedures are to experimental science” ([91], p6). Git is a decentralized open source version control system that’s useful regardless of whether a project is independent or collaborative [92]. GitHub works in conjunction with Git to provide a powerful structured system to organize and manage components of a project for others and your future self. A growing number of scientists have research programs based in GitHub, which has become a revolutionary tool for productive team science and distributed development efforts [86,93]. As you may expect, Git coupled with GitHub is the version control duo of choice among serious R package developers [31]. Thus, if the package you’re interested in using is among the thousands hosted on GitHub, this is evidence that the developer is at least committed to a logical, open, and reproducible workflow, suggestive of a more carefully designed tool.

Lastly, developers often implement CI in conjunction with a version control hosting platform like GitHub. For R package developers, CI typically involves incorporating GitHub Actions to configure workflows within their GitHub repository, or utilizing an outside service like Travis CI, to run automated tests (for instance, unit tests, CRAN checks) on package code every time they push new contributions and changes to their (potentially shared) GitHub repository [31,38]. Just as you’re happy to know that a manufacturer guarantees their product is a unified realization of many working parts, you should be impressed when a package is set up to use CI. This is particularly important for packages with a multiperson development team, and also an indicator of quality for single-author packages. Use of CI is a strong sign that the components of the package are regularly checked to ensure that code passes tests and the package builds without failures [94].

## Rule 9: Put it to the test

If you’re unable to decide whether to use a package based on prior Rules, test it out. Similarly, if you have narrowed your options, work with each to highlight differences. Exploring each package and engaging in trial and error using your skills in context of your goal will illuminate technical details and solidify any of your doubts.

At this point, what you’ve learned about the package should be quite helpful. If the package has a vignette, this is a great place to start experimenting with code. Vignettes include many common data science problems with solutions; you can run the code examples, tweak them, and compare the outputs. First, try some examples with the example data provided by the package, then see if you can translate that to working with your own data. Sometimes, package functions will require inputs to be in a specific data type (for instance, a dataframe, or a certain type of S4 object) or to have a specific format (for instance, if it’s in a dataframe, to have certain columns with certain names). By moving from the example data to your own, you can gauge the effort it will take to transform your data to fit these formats. You can also be on the lookout for whether certain characteristics of your data (for instance, fields with missing values) cause complications when using the package’s functions. You can continue by looking through the collection of help files for the package. Help files often include executable examples, and you can again start by testing these with the example data provided by the package, then move on to trying them with your own data and within your own workflow.

Sometimes packages don’t interact well with other packages. You should therefore test the interoperability of all the packages you want to use. There are some packages that are masterful at doing what they’re made to do, yet incompatible with other packages. Such packages might, for example, use S3 or S4 objects, which are 2 main approaches developers use to implement object-oriented programming in R. Many packages for spatial analysis, as well as those from Bioconductor, tend to use S4 objects to represent data [3]. On the other hand, the tidyverse, a unified suite of packages with an grammatical structure employed within a “pipeline,” expects dataframe objects [79]. Because of this, when you’re working in the tidyverse, you may need to take extra steps to extract components from S3/S4 objects to reframe the data into a dataframe. Some packages already exist to help with this interfacing task between S3/S4 objects and tidyverse tools. The broom package, and the bioinformatics analog, biobroom, aim to alleviate these disruptions by converting “untidy” objects in S3/S4 objects into “tidy” data in dataframes, thereby making it easier to integrate statistical functions into the structure of the tidyverse workflow [11,79,95]. Similarly, the caret package, as well as the parsnip package within the newer tidymodels framework of packages, facilitate interoperability for machine learning packages by providing a uniform interface for modeling with various algorithms from different packages that would otherwise have independent syntax [13,59,96].

## Rule 10: Develop your own package

What if you can’t find a package to solve your data science problem? Rather than exclusively being a user of packages, you can create them—more easily than you may think [31].

If you decide to write your own R code to perform your task (see Rule 1), a natural next step is to wrap this code into a function that can be reused; an R package is basically a collection of one or more of these functions. The reasons why you might want to create a package are abundant: necessity, innovation, standardization, automation, specialty, containment, organization, sharing, collaboration, extensibility, and more. Whatever your motivation, packages are simply sets of tools; you can create a package out of any collection of specialty functions. A key tenet of efficient programming is the “DRY” principle—Don’t Repeat Yourself. Functions help with this and are warranted when you copy and paste code while making slight modifications after each iteration [31]. You should use a function when you want to replace repetitive code with a defined, compact piece of code defined only once in your script, which you can call throughout in shorthand. Since R packages are just sets of these functions, they’re not solely reserved for large-scale, comprehensive methods; rather, package development can also help you learn how to apply proper coding techniques to writing functions and documentation with reproducibility and collaboration in mind [97]. Packages need not be formal nor entirely cohesive. For instance, personal R packages such as Hmisc and broman are comprised of miscellaneous functions that the creator has developed and frequently uses [98,99].

While personal R packages are virtually unbounded, we’ll describe some of their immediate uses. If you often do the same specific, discipline-oriented data analysis tasks—for which either a package doesn’t exist or you don’t want to rely one that does (see Rule 1)—a personal R package could help you automate some redundant aspects of your work. You can even connect components of other tools you’re using that wouldn’t otherwise play nicely together. For example, if you’re work largely involves packages that output S3/S4 object types, but you want to use standard ggplot2 tools to visualize the output, you’ll encounter a problem because functions in ggplot2 expect a dataframe as the standard input, not these more complex object types [14]. In this case, you might want to write some of your own functions that extract what you need from the S3/S4 objects and convert them into a dataframe input type so they’re compatible with ggplot2. Interestingly, this type of occurrence is the precise motivation behind the widely used broom package [11,100]. You can also create visualization tools that are tailored to your research and include them in your personal R package. If you frequently design a certain type of plot with your data, you can encode that process into a function, even if it relies heavily on functions from other packages. For instance, this could happen if you implement particular tweaks whenever you make scatterplots with your data; many R users build custom themes within ggplot2 for purposes like personal or professional branding, which can be wrapped into functions and contained in a personal R package [14,94].

Although you may not anticipate that anyone else will use your tools, following best practices for package development is still a good idea. Even if you’re the only person who will use your package, be kind to your future self—as a consumer of shared packages, you know the inherent benefits of robust software development relative to the quality of code, data, documentation, versions, and tests [85]. Take advantage of existing resources when coding your package. For example, there are R packages that aid in package development (for instance, devtools, usethis, testthat, roxygen2, rlang, drat) [39,45,88,101–103]—while these packages can provide industrial-strength support for developing large and complex packages, they also prove useful even in developing small, private packages. They make it easier and more efficient, for example, to write help documentation and unit tests for functions in your package. RStudio’s IDE has a framework called “Projects,” which can help you establish an organized framework of directories within an R Project (.Rproj)—specifically designed for creating R packages—that’s straightforward to navigate throughout the package development process. Tips on using many of these package development tools are available through resources authored by expert R developers, like R Packages by Hadley Wickham [31], while the official resource on writing packages to share through CRAN is the manual, Writing R Extensions [104].

Regardless of whether you’re writing a package to share, or one for your own use that you don’t plan to publish through a repository, consider using version control. Among the numerous reasons why version control will positively impact your workflow, you’ll be able to easily share your project with collaborators, at any point in time. If you decide to collaborate on a package, even if you just intend for the package to remain a resource among your group, the Project framework is compatible with distributed development—a feature that couples well with version control and online platforms for version controlled repositories, like GitHub. Version control also helps you maintain a clean directory of code without limiting the process of making changes to files. All changes are trackable and rewindable so earlier versions are restorable. You can create offshoots of your main project (branches) to try new things and incorporate some (or all) of the changes into the main branch. As you work, keep in mind that within the open source R community, there’s no lack of effective organizational frameworks to reference; in fact, repositories for many exemplary packages are readily available on GitHub.

## Conclusions

The open source framework is the heartbeat of R; it sustains and enlivens the growth and improvement of the R ecosystem. Anyone, whether they’re motivated to propel a research program or interested in learning from data in other contexts, can be an R user—without significant barriers to entry. A number of R users don’t stop there, however. They become R makers by adding their own contributions, so not only they, but new and existing users and makers, can do more in R. This plays an essential role in the life cycle of R’s beloved shared tools.

R packages are a defining feature of the language insofar as many are robust, user-friendly, and richly extend R’s core functionality. Some of the most prominent R packages are a result of the developer abstracting common elements of a data science problem into a workflow that can be shared and accompanied by thorough descriptions of the process and purpose. In this way, R packages have effectively transformed how we interact with data in the modern day in, perhaps, a more impactful manner than several revered contributions to theoretical statistics [1]. Packages greatly enhance the user experience and enable you to be more efficient and effective when working with data, regardless of prior experience.

Nonetheless, the sheer quantity and potential complexity of available R packages can undermine their collective benefits. Finding and choosing packages, particularly for beginners, can be daunting and difficult. Even so, R users of all abilities may, at some point, struggle to sift through the tools at their disposal and wonder how to distinguish appropriate usage. These 10 simple rules for navigating the shared code in the R community are intended to serve as a valuable page in your computing toolbox—one that will evolve into intuition and yet remain a reliable reference.
